# Study of clinical traits and systemic immune inflammation index assessments in patients with endogenous endophthalmitis over the last ten years

**DOI:** 10.1186/s12886-023-03266-9

**Published:** 2024-01-05

**Authors:** Rui Niu, Zhongyang Yan, Yanhui Wang, Yalin Li, Wei Feng, Jianan Liu, Lifei Wang

**Affiliations:** Hebei Eye Hospital, Hebei Provincial Key Laboratory of Ophthalmology, Hebei Provincial Clinical Research Center for Eye Diseases, Xingtai, Hebei Province, China

**Keywords:** Endogenous endophthalmitis, Systemic immune inflammation index, Visual acuity

## Abstract

**Purpose:**

The clinical aspects and prognosis of eyes with endogenous endophthalmitis were compared over the last ten years. The occurrence and progression of endophthalmitis are linked to the systemic immune inflammation index (SII) and clinical features.

**Methods:**

The study comprised patients with endogenous endophthalmitis and 64 patients without endophthalmitis who were treated at Hebei Province Eye Hospital in the last ten years. According to the prognostic visual acuity, patients with endophthalmitis were split into two groups: Group A and Group B. Underlying disease (hypertension, diabetes, tuberculosis), infection risk (liver abscess, urinary tract infection, and recent abdominal surgery), signs and symptoms, and complete blood count were among the evaluation parameters (neutrophil count, lymphocyte count, monocyte count, platelet count, red blood cell distribution width). The NLR, PLR, MLR, and SII values were calculated. A nonparametric test was used to examine the clinical features and complete blood count results of patients in each group. To determine the parameters linked to endophthalmitis progression, researchers used principal component and ordinal logistic regression analyses.

**Results:**

The study comprised a total of 25 eyes and 22 individuals with endogenous endophthalmitis. Infectious bacteria included Staphylococcus aureus, Micrococcus luteus, Staphylococcus hemolyticus, and so on. The visual acuity of the affected eye ranged from 2.7 (1.55, 2.7) LogMAR to 1.22 (0.6, 2.7) LogMAR during the 6-month to 8-year follow-up period. The neutrophil, monocyte, and PLT counts, NLR, PLR, and SII values and other markers were considerably higher in Groups A and B than in the control group. The likelihood model of the SII and sex, age, onset time, diabetes, hypertension, monocyte count, and red blood cell distribution was the best, and its increase was strongly connected with the occurrence and progression of endophthalmitis, according to ordinal regression analysis.

**Conclusion:**

Patients with endophthalmitis had significantly higher blood neutrophil, monocyte, and PLT counts and SII, NLR, PLR, and MLR values. The SII can be employed as a biomarker for predicting endophthalmitis severity and prognosis.

**Supplementary Information:**

The online version contains supplementary material available at 10.1186/s12886-023-03266-9.

## Introduction

Endogenous endophthalmitis (EE) is a less common but serious intraocular infection caused by the spread of infectious agents from distant foci of infection to the eye through the bloodstream [1]. The condition has a very poor visual prognosis, and many patients have underlying or related immunosuppression [2]. Endophthalmitis has a short clinical course, hence early diagnosis and treatment are critical for visual recovery. However, delayed onset and therapy are issues that must be addressed, and current diagnosis and treatment approaches must be improved. The progression of endophthalmitis is linked to underlying disorders and acute infection, and standard blood tests are accurate and fast.

The Systemic Immune Inflammation Index (SII) is a new predictive scale for immune inflammation based on neutrophil, lymphocyte, and platelet counts. It is a prognostic indicator and an independent predictor of a variety of diseases, such as cancer, coronary artery disease, autoimmune disease, and infectious disease [3–6]. The SII has been linked to primary open-angle glaucoma, keratoconus, and dry eye disease in the field of ophthalmology [7–9]. At the same time, the neutrophil–lymphocyte ratio (NLR), platelet-lymphocyte ratio (PLR), and monocyte-lymphocyte ratio (MLR) have been used as important metrics of systemic inflammation in many conditions, such as tumors, blood disorders, and chronic inflammation, with incidence and progression being important factors to consider in evaluation [10–13].

As a result, the SII, NLR, and PLR are used in conjunction with characteristics such as underlying disorders, infection risk, symptoms, and signs as evaluation factors. Models were built based on the relationship between the occurrence and progression of diseases. To better understand the predictors, we laid the groundwork for investigating factors in the evaluation of endogenous endophthalmitis.

## Methods

### Study design

The study included 64 patients without endophthalmitis with routine visits who were matched according to quantity and patients with EE who visited Hebei Provincial Eye Hospital in the last ten years. Patients with a history of recent ophthalmic surgery (within 1 year of the study), a history of ocular trauma, and primary eye infection were excluded (e.g., blister infection or keratitis). This study was approved by the Ethics Committee of Hebei Provincial Eye Hospital (2022LW01) and conformed to the principles of the Declaration of Helsinki. All subjects signed informed consent forms.

To assess ocular conditions, all eyes were tested for visual acuity and performing a fundus examination by using a slit lamp microscope. Underlying disease (hypertension, diabetes, tuberculosis), infection risk (liver abscess, urinary tract infection, and recent abdominal surgery), signs and symptoms, complete blood counts (neutrophil count, lymphocyte count, monocyte count, platelet count, red blood cell distribution width), and SII, NLR, PLR, and MLR values were some of the evaluation parameters in this study [9]. All patients with endophthalmitis were followed up and divided into two groups based on their visual acuity prognosis: Group A had a good visual prognosis (visual acuity > 1.30 LogMAR), Group B had a poor visual prognosis (visual acuity < 1.30 LogMAR or a 10° visual field surrounding blindness and eye enucleation with central gaze). The control group included patients without endophthalmitis who had routine visits [14]. The LogMAR visual acuity chart was used to convert the visual acuity findings. In individuals with binocular illness, a follow-up analysis was performed based on the state of the heavier eye.

### Statistical analysis

SPSS 26.0 was used for all analyses. The results were checked for a normal distribution by the Shapiro‒Wilk test. Nonparametric Tests Quantitative variables are described as medians. The chi-square test or Fisher's exact test was used to compare categorical variables. Continuous independent variables were subjected to principal component analysis. Ordinal logistic regression analysis and a parallelism test were used to examine the relevant independent variables.

## Results

### Characteristics of patients with endophthalmitis

There were 22 patients and 25 eyes with endogenous endophthalmitis, accounting for 3.31% of all endophthalmitis cases. There were 9 men and 13 women in this group. The average age was 53 years (30, 66). The mean time to the start of the study was 6 (4, 10) days, and all patients complained of diminished visual acuity. There were four cases of diabetes, six cases of hypertension, three cases of pulmonary tuberculosis, and two cases of recent abdominal surgery among the patients.

**Fig.1:**
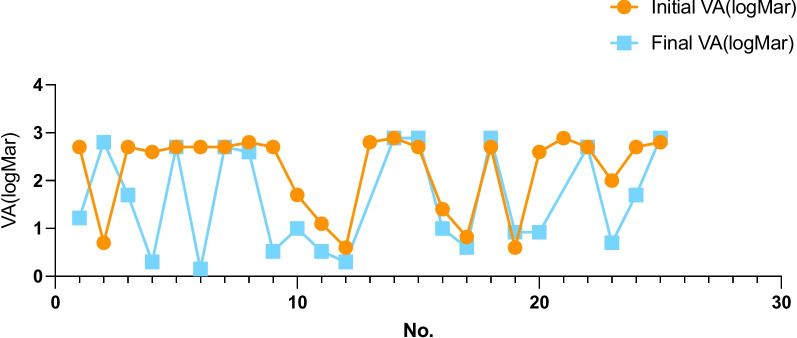
shows the changes of visual acuity at admission and final follow-up in patients with endogenous endophthalmitis: the visual acuity of patients with endogenous endophthalmitis changed significantly after treatment compared with before treatment (*P* = 0.008). The number of the affected eye on the abscissa and the visual acuity on the ordinate

Among the gram-positive pathogenic bacteria were 1 case of *Staphylococcus aureus*, 1 case of *Micrococcus luteus*, 1 case of *Staphylococcus hemolyticus*, 1 case of *Staphylococcus epidermidis*, and 1 case of *Bacillus cereus*; among the gram-negative bacteria were 1 case of Klebsiella and 1 case of *Serratia marcescens*; and among the fungi were 1 case of Mucor, 1 case of Candida, and 1 case of *Fusarium oxysporum*. Vitreous opacity was seen in all 25 eyes, with corneal edema in 10 eyes, hyphema in 9 eyes, lens opacity in 12 eyes, and retinal detachment in 8 eyes. Nine patients underwent vitreous injection, 5 patients underwent vitrectomy and vitreous injection, 1 patient underwent lens resection with vitrectomy and vitreous injection, 1 patient underwent lens resection, 1 patient underwent enucleation, 1 patient underwent evisceration, and 1 patient had given up treatment. The study follow-up period ranged from 6 months to 8 years. The vision of the damaged eye was examined for any changes. Four of the 22 patients could not be followed up, and the data of patients who could not be followed up were recorded according to their discharge vision. Three patients died of illness, using the latest follow-up as the time point for efficacy judgment. The final visual acuity ranged from 1.22 (0.6, 2.7) LogMAR to 2.7 (1.55, 2.7) LogMAR (*P* = 0.008) (Table 1) (Fig. 1).

**Table 1:** Demographic information of the patients

NO	Age	Sex	Affected eye	Onset time	Underlying disese	Pathogen isolated	Performance	Type of Surgery	Initial VA	Final VA
1	42	M	OD	6	-	Staph. aureus	PCO、RD	PPV	2.70	1.22
2	66	M	OD	10	-	Fungus Mucor	Corneal Edema, Hypopyon, Cat, RD	PPV + IVI	0.70	2.80
3	40	M	OS	5	HTN	-	Cat	-	2.70	1.70
4	7	M	OD	2	-	-	Corneal Edema, Hypopyon	IVI	2.60	0.30
5	56	M	OS	10	hepatapostema	-	Corneal Edema, Hypopyon, Cat	PPV + IVI	2.70	2.70
6	30	M	OD	4	-	-	Corneal Edema, RD	IVI	2.70	0.15
7	52	F	OD	17	tuberculosis	-	Cat, RD	PPV	2.70	2.70
8	60	F	OS	5	HTN	M.luteus	Corneal Edema, Cat	PPL	2.80	2.60
9	37	F	OD	1	Tuberculosis, Oral Prednisone	-	-	PPV	2.70	0.52
10	30	F	OD	10	Abdominal operation history	candida	-	PPV + IVI	1.70	1
			OS				-	PPV + IVI	1.10	0.52
11	32	F	OS	10	Urethral calculi, DM	S.marcescens	-	IVI	0.60	0.30
12	54	F	OD	10	DM, HTN, Urethral calculi	-	-	Enucleation	2.80	-
13	67	F	OS	10	DM, HTN	Bacillus cereus	Corneal Edema, Cat, RD	IVI	2.89	2.89
14	68	F	OD	3	HTN	K.pneumoniae	Corneal Edema, Hypopyon, Cat, RD	PPV + IVI	2.70	2.89
15	55	F	OD	7	-	F. oxysporum	PCO	IVI	1.40	1
			OS				PCO, MH	IVI	0.82	0.60
16	77	F	OD	13	Bronchiectasis infection	-	Corneal Edema, Hypopyon, IOL	Reject	2.70	2.89
			OS				Cat		0.60	0.92
17	56	F	OD	3	DM	-	Cat	IVI	2.60	0.92
18	73	F	OS	6	DM, HTN	MRSH	Corneal Edema, Hypopyon, Cat, RD	Evisceration	2.89	-
19	69	M	OD	2	Abdominal operation history	-	Corneal Edema, Hypopyon, Cat, RD	IVI	2.70	2.70
20	7	F	OS	4	-	-	-	IVI	2	0.70
21	24	M	OS	9	-	S. epidermidis	Hypopyon	PPV + PPL + IVI	2.70	1.70
22	5	M	OS	5	-	-	Hypopyon, Cat	PPV	2.80	2.89

"-" in Type of Surgery stands for local anti-inflammatory treatment

### Patient characteristics linked to the development and progression of endophthalmitis

There were 64 patients in the control group; 9 and 13 patients with endogenous endophthalmitis were included in Group A and Group B, respectively. In terms of onset time, sex, age, diabetes, hypertension, pulmonary tuberculosis, liver abscess, urinary tract infection, abdominal surgery history, corneal edema, hyphema, lens opacity, retinal detachment, and so on, the control group, Group A, and Group B had significantly different expression patterns (all *p* < 0.05) (Table 2).

**Table 2:** Comparison of characteristics of patients with group normal, A and B

(Variables)	Control	A	B	*P*
No.of patients	64	9	13	
Onset time	180(60,730)	4(2.5,8.5)	9(5,10)	** < 0.001**
Gender	28(43.75)	3(33.33)	6(46.15)	**0.002**
Age	58.5(43,72.75)	32(18.5,48.5)	60(46, 68.5)	**0.014**
Systemic disease				
Diabetes	11(17.19)	2(22.22)	3(23.08)	**0.005**
Hypertension	18(28.13)	0(0)	6(46.15)	** < 0.001**
Tuberculosis	1(1.56)	1(11.11)	2(15.38)	**0.001**
Liver abscess	0(0)	0(0)	1(7.69)	**0.012**
Urinary tract infection	0(0)	1(11.11)	1(7.69)	**0.002**
Abdominal surgery	0(0)	1(11.11)	1(7.69)	**0.002**
Ocular symptoms				
Corneal edema	1(1.56)	2(22.22)	9(69.23)	** < 0.001**
Hypopyon	0(0)	1(11.11)	(69.23)	** < 0.001**
Cataract	46(71.88)	3(33.33)	10(76.92)	** < 0.001**
Retinal detachment	5(7.81)	2(22.22)	8(61.54)	** < 0.001**

Important findings (*P* < 0.05) are shown in bold. The value of continuous variable is the median (P25, P75), and the dummy variable is the number of cases and proportion (%)

### Analysis of blood cells associated with the development and progression of endophthalmitis

Endophthalmitis patients had significantly higher neutrophil counts, monocyte counts, PLT counts, and SII, NLR, PLR, and MLR values than controls (*p* < 0.001; *p* < 0.001; *p* < 0.001; *p* < 0.001; *p* < 0.001; *p* < 0.01; *p* < 0.001). In patients with endophthalmitis in Group B, the neutrophil count, monocyte count, PLT count, and SII, NLR, and MLR values were all considerably higher (*p* < 0.001; *p* < 0.001; *p* < 0.01; *p* < 0.001; *p* < 0.001; *p* < 0.01) (Table 3).

**Table 3:** Blood analysis of participants

(10^3^/uL)	Control / All Endophthalmitis(*n* = 64/22)	P	Control / Group A(*n* = 64/13)	*P*	Group A / Group B(*n* = 9/13)	*P*
Neutrophil	3.09(2.06,3.91)/6.74(3.85, 8.66)	** < 0.001**	3.09(2.06,3.91)/ 7.84(6.21, 11.04)	** < 0.001**	4(2.81,6.57)/ 7.84(6.21, 11.04)	0.07
Lymphocyte	1.82(1.42,2.35)/ 1.81(1.49, 2.44)	0.92	1.82(1.42,2.35)/ 1.8(1.57, 2.36)	-	1.9(1.27, 2.49) / 1.8(1.57, 2.36)	-
Monocyte	0.29(0.25,0.38)/ 0.48(0.39, 0.56)	** < 0.001**	0.29(0.25,0.38)/ 0.5(0.39, 0.57)	** < 0.001**	0.4(0.33, 0.54) / 0.5(0.39, 0.57)	0.90
PLT	222.5(182.25, 264.25)/ 307(233.75, 412)	** < 0.00**	222.5(182.25, 264.25)/ 300(232.5, 442)	**0.008**	314(253, 381.5) / 300(232.5, 442)	1
SII	385.19(258.65, 569.27)/ 1198.82(549.08, 2018.82)	** < 0.001**	385.19(258.65, 569.27)/ 1687.16(863.57, 2636.62)	** < 0.001**	790.38(324.18, 1801.32) / 1687.16(863.57, 2636.62)	0.28
NLR	1.89(1.25, 2.58)/ 3.93(1.85, 6.33)	** < 0.001**	1.89(1.25, 2.58)/ 5.05(3.06, 7.80)	** < 0.001**	1.92(1.09, 5.25) / 5.05(3.06, 7.80)	0.09
PLR	115.39(95.77, 149.11)/ 178.66(116.18, 266.90)	**0.004**	115.39(95.77, 149.11)/ 180.52(101.32, 274.18)	0.07	165.26(115.85, 279.14) / 180.52(101.32, 274.18)	1
MLR	0.16(0.12,0.21)/ 0.29(0.17, 0.36)	** < 0.001**	0.16(0.12,0.21)/ 0.31(0.20, 0.37)	**0.001**	0.18(0.15, 0.39) / 0.31(0.20, 0.37)	0.88
Red Cell Distribution Width (%)	12.6(12.13, 13)/ 12.85(12,13.83)	0.47	12.6(12.13, 13)/ 13(12.4, 13.95)	-	12.1(12, 15.3)/ 13(12.4, 13.95)	-

“-”stands for nonparametric test meaningless. The value is represented by M (P25, P75)

### Principle component analysis

The neutrophil count, lymphocyte count, platelet count, monocyte count, red blood cell distribution, age, and onset time were all subjected to principal component analysis (KMO > 0.6, *P* < 0.05). The cumulative contribution rate for the 3 main components was 73.90%. The first principal component, Z1, had a higher factor load in the platelet count, monocyte count, and age than the other components, and it was thought that this component primarily reflected the inflammatory condition. The factor load of the second principal component, Z2, was larger in the neutrophil and lymphocyte count, and it was believed that this principal component mainly reflected the changes in infection-related indices. In terms of the red blood cell distribution and onset time, the third major component, Z3, had a higher factor load. This main component was thought to primarily reflect information on these factors (Table 4). However, it is worth noting that as shown in Table 4, although the PCA principal components have a certain degree of discrimination in Z1, the discrimination is not very good, so there is an overlap between Group A and the control group rather than Group B (Fig. S1) (Table S1).

**Table 4:** Factor loading matrix

index	Z1	Z2	Z3
neutrophils	0.513	0.715	-0.103
lymphocytes	0.472	-0.674	-0.326
Platelet count	0.769	0.055	-0.176
monocytes	0.747	0.362	-0.144
RDW-CV	0.404	0.32	0.713
age	-0.653	0.571	-0.036
Onset time	0.237	-0.496	0.588

### Ordinal logistic regression analysis evaluation

The association among the SII, NLR, PLR, and MLR values, reduced dimension primary components and endophthalmitis development was investigated. For sex, age, onset time, diabetes, hypertension, monocyte count, red blood cell distribution, and SII, NLR, PLR, and MLR values, compared with control, logistic regression analysis was used. Ordinal logistic regression analysis was performed on the dimension-reduced principle components, as well as sex, diabetes, and hypertension. The cumulative probability model was statistically significant (*P* < 0.05). The model built using the SII to evaluate the occurrence and development of endophthalmitis was deemed the best when the AIC value (73.34, 75.90, 79.15, 78.73, 104.77) of the probability model involving SII, NLR, PLR, and MLR values and the principal components was compared to the P value (0.02, 0.04, 0.21, 0.16, 0) of each index. The OR values and 95% confidence intervals of the SII and onset time in this model were 1.002 (1–1.003) and 0.911 (0.833–0.996), respectively. This result indicated that when the patient's condition worsened by one grade, the SII increased by 1.002 times, and the onset time increased by 0.91 times. The SII and onset time had a significant effect on the occurrence of endophthalmitis (Table 5).

**Table 5:** Ordinal logistic regression of potential biomarkers in relation to the occurrence and progression of EE

**Predictors**			**Predictors**			**Predictors**			**Predictors**			**Predictors**		
	**OR (95% CI)**	***P *Value**		**OR (95% CI)**	***P *Value**		**OR (95% CI)**	***P *Value**		**OR (95% CI)**	***P *Value**		**OR (95% CI)**	***P *Value**
SII	1.002 (1-1.003)	**0.02**	NLR	1.65(1.03-2.63)	**0.04**	PLR	1.01(1.00-1.02)	0.21	MLR	0(0.000000012-19.21)	0.16	PCA	6.30(2.98-13.33)	**0**
gender	1.79(0.28-11.37)	0.54	gender	2.91(0.42-20.04)	0.28	gender	1.70(0.30-9.59)	0.55	gender	1.68(0.27-10.25)	0.58	gender	0.61(0.18-2.08)	0.43
age	0.38(2.05-1.30)	0.82	age	0.99(0.95-1.03)	0.53	age	1.004(0.97-1.04)	0.85	age	1.01(0.97-1.06)	0.55	DM	0.39(0.09-1.73)	0.21
Onset time	0.91(0.83-1.00)	**0.04**	Onset time	0.92(0.84-1.00)	**0.04**	Onset time	0.91(0.84-0.99)	**0.03**	Onset time	0.90(0.82-0.98)	**0.02**	HTN	0.78(0.21-2.89)	0.71
DM	1.07(0.12-9.20)	0.95	DM	1.26(0.16-10.27)	0.83	DM	1.12(0.15-8.65)	0.92	DM	0.87(0.12-6.51)	0.90			
HTN	0.55(0.08-3.62)	0.53	HTN	0.53(0.08-3.36)	0.50	HTN	0.51(0.08-3.04)	0.46	HTN	0.51(0.08-3.38)	0.48			
monocytes	262.11(0.33-206901.02)	0.10	monocytes	491.00(0.52-460545.16)	**0.08**	monocytes	3747.03(5.15-2725723.35)	**0.01**	monocytes	640417.38(35.711-11484898424.80)	0.01			
RDW-CV	0.79(0.54-1.16)	0.23	RDW-CV	0.82(0.56-1.20)	0.31	RDW-CV	0.89(0.62-1.278)	0.52	RDW-CV	1.14(0.79-1.62)	0.49			

## Discussion

Endogenous endophthalmitis, which accounts for 2% to 15% of all endophthalmitis cases, is a dangerous intraocular infection caused by the hematogenous spread of bacterial germs [15–17]. Diabetes, cancer, urinary tract infections, intravenous drug misuse, an immunocompromised status, and other potentially life-threatening systemic sources of infection or risk factors for infection are linked to 56 percent to 68 percent of endophthalmitis cases [15, 18, 19]. Endogenous endophthalmitis can be caused by bacteria or fungi, mainly gram-positive bacteria such as Staphylococcus and Streptococcus, gram-negative bacteria such as Klebsiella [18, 19], and fungi such as Candida [20]. In this study, only some patients had positive aqueous humor cultures, mainly for gram-positive bacteria and fungi. Although endophthalmitis is closely related to bacteria and fungi, the positive intraocular culture rate in patients with endogenous endophthalmitis is usually low, at only 14–43% [20]. Given this low diagnostic rate, physicians generally base treatment on their clinical experience [20]. However, it was not comprehensive to focus on a certain index to reflect the disease.

In addition, when comparing the visual acuity of patients before and after therapy, it was discovered that the improvement in visual acuity was lower in patients with endophthalmitis after treatment, which was not what was expected. This finding is in line with earlier research on endophthalmitis and visual acuity [21]. The reason for consideration is that the current diagnosis and treatment methods have difficulty controlling the progression of endophthalmitis early. The discovery of more illness-influencing factors will aid in the identification of disease targets for early diagnosis and treatment. As a result, in this study, we attempted to use some biomarkers, such as the SII, NLR, and PLR, as well as analyze the clinical characteristics of patients to determine which factors are linked with the progression of endophthalmitis [22].

The SII is a good biomarker of the local immune response and systemic inflammation, and it can be used to predict outcomes. It has been confirmed as a good biomarker for a variety of tumors, such as hepatocellular carcinoma, esophageal cancer, colorectal cancer and small cell lung cancer [23–25]. There were substantial differences between the endophthalmitis patients in Group B and individuals in the control group in terms of the neutrophil count, the monocyte count, the PLT count and SII, NLR, PLR and MLR values. Endophthalmitis patients also exhibited considerably greater neutrophil counts, monocyte counts, PLT counts, and SII, NLR, PLR, and MLR values than individuals in the control group, indicating that inflammatory markers accurately depicted the inflammatory response in the group with disease and may be utilized as a diagnostic tool. Inflammatory markers are some of the indications used to help diagnose the course of inflammation.

However, it was not comprehensive to focus on a certain index to reflect the disease. Therefore, a probability model was further constructed with variables such as sex, age, onset time, diabetes, hypertension, monocyte count, and red blood cell distribution. It was found that the SII and sex, age, onset time, diabetes, hypertension, monocyte count, and red blood cell distribution were the most accurate logistic models. Therefore, it is believed that the SII and clinical characteristics are related to the occurrence of endogenous endophthalmitis, which is the most suitable for evaluating the possibility of the occurrence and development of endogenous endophthalmitis in patients. Furthermore, both the SII and onset time have an impact on endophthalmitis diagnosis. The SII value rises exponentially as the severity of endophthalmitis increases.

The SII is better as an evaluation index, and whether it is related to more fusion indices and whether multiple indices are more conducive to evaluating disease models needs to be explored. Therefore, neutrophil, lymphocyte, platelet, and monocyte counts, red blood cell distribution, age, and onset time were all subjected to principal component analysis. After dimensionality reduction, the comprehensive score was combined with characteristics including sex, diabetes, and hypertension to create a probability model. The model's accuracy was lower than that of the SII probability model. As a result, the SII is believed to be a better marker for early diagnosis and therapy. The structure of the factor loading matrix generated from the principal component analysis revealed that the endogenous endophthalmitis principal components had a specific orientation in representing inflammation in vivo, which could lead to specific ideas for further research and study of inflammatory markers.

There were 9 males and 13 females in this study, and the sex and age distributions were significantly different. The shorter mean time to onset is consistent with other literature [18]. The patients had diabetes, hypertension, pulmonary TB, recent abdominal surgery, liver abscess, urinary tract stones, long-term hormone use, and other systemic infection sources and risk factors. Endogenous endophthalmitis is commonly caused by diabetes and cancer, and liver abscesses are a major source of infection in Asian countries [19, 20, 26]. Diabetes is thought to affect the blood‒retinal barrier by altering blood vessel structure [15]. Pathogens pass the blood-eye barrier, fight off the body's immune system, and grow in the eye, causing endogenous endophthalmitis [20]. Only a few pathogens in the vitreous cavity can cause endophthalmitis because the blood-eye barrier is well immunized in most cases. In unprovoked young adults, however, more virulent bacteria such as *Staphylococcus aureus* can disrupt the retinal barrier and cause endogenous endophthalmitis [27].

In this study, 9 patients recovered well, and the treatment methods included vitrectomy and intravitreal injection. Thirteen patients had poor recovery, and the treatment methods included vitrectomy, intravitreal injection, lens resection, and local anti-inflammatory treatment. Relevant studies suggest that vitrectomy can improve the visual acuity of patients [28]. This study discovered no significant differences, which were attributed to the onset time, the degree of inflammation progression, bacterial invasiveness, and the small number of positive samples. There are some drawbacks to this study. First, because it was a retrospective study, this study may be prone to selection bias, as well as detection and analytical bias. Second, the sample size of the group with endogenous endophthalmitis was modest, and no significant variations in systemic features, such as diabetes and hypertension, were detected during the illness course. Therefore, patients with the same surgical procedure were limited, and no groupings were compared.

Endophthalmitis is influenced by a variety of factors, including underlying disorders and infection risk, and has distinct symptoms and signs, although it is difficult to diagnose and has a poor prognosis. Alterations in the inflammatory state in patients with endophthalmitis were observed by analyzing the neutrophil, monocyte, and PLT counts and the SII, NLR, PLR, and MLR values. The SII was found to be a better predictor of endophthalmitis progression, laying a foundation for further exploration of diagnostic indicators of endophthalmitis and the prediction of disease progression.

### Supplementary Information

**Additional file 1:** **Fig. S1.** shows that the ellipse represents the core area grouped by the default 68% confidence interval, the correlation ring (ie the circle, representing the correlation), and the arrow represents the principal component loading. **Table S1.** Spearman correlation study on blood routine test indicators, SII, NLR, PLR, MLR, age, and onset time.

## Data Availability

The data used to support the findings of this study are available from the corresponding author upon request.
