# Supplementary material for: Study of clinical traits and systemic immune inflammation index assessments in patients with endogenous endophthalmitis over the last ten years
Source: BMC Ophthalmol. 2024 Jan 5;24:11. doi: 10.1186/s12886-023-03266-9 (PMC10768133; doi:10.1186/s12886-023-03266-9)
Supplement: Supplementary file 1 — Additional file 1: Fig. S1. shows that the ellipse represents the core area grouped by the default 68% confidence interval, the correlation ring (ie the circle, representing the correlation), and the arrow represents the principal component loading. Table S1. Spearman correlation study on blood routine test indicators, SII, NLR, PLR, MLR, age, and onset time. [file 12886_2023_3266_MOESM1_ESM.docx]

**Fig.S1** shows that the ellipse represents the core area grouped by the default 68% confidence interval, the correlation ring (ie the circle, representing the correlation), and the arrow represents the principal component loading.

**TableS1.** Spearman correlation study on blood routine test indicators, SII, NLR, PLR, MLR, age, and onset time

| Variables | Neutrophil | Lymphocyte | PLT | Monocyte | Red Cell Distribution Width (%) | NLR | PLR | MLR | SII | age | Onset time |
| --- | --- | --- | --- | --- | --- | --- | --- | --- | --- | --- | --- |
| Neutrophil | 1 |  |  |  |  |  |  |  |  |  |  |
| Lymphocyte | -0.178 | 1 |  |  |  |  |  |  |  |  |  |
| PLT | .354** | .288** | 1 |  |  |  |  |  |  |  |  |
| Monocyte | .510** | 0.143 | .487** | 1 |  |  |  |  |  |  |  |
| Red Cell Distribution Width (%) | .250* | -0.17 | 0.083 | 0.164 | 1 |  |  |  |  |  |  |
| NLR | .811** | -.680** | 0.098 | .319** | .309** | 1 |  |  |  |  |  |
| PLR | .392** | -.638** | .495** | 0.166 | 0.191 | .660** | 1 |  |  |  |  |
| MLR | .539** | -.681** | 0.062 | .577** | .212* | .801** | .612** | 1 |  |  |  |
| SII | .861** | -.473** | .464** | .447** | .294** | .907** | .777** | .715** | 1 |  |  |
| age | 0.091 | -.466** | -.393** | -.232* | -0.093 | .318** | 0.118 | .269* | 0.144 | 1 |  |
| Onset time | -.352** | 0.118 | -0.128 | -.293** | 0.013 | -.339** | -.224* | -.334** | -.353** | 0.04 | 1 |

*p < 0.05; **p < 0.01; *** p<0.001; Correlation coefficients greater than 0.6 represent covariance and are indicated in bold type.
